# Pancreatic Cancer-Targeting Cascade Nanoamplifier Enables Self-Replenishing H_2_O_2_ Generation and Autophagy Disruption in Chemodynamic Therapy

**DOI:** 10.3390/pharmaceutics17091201

**Published:** 2025-09-16

**Authors:** Jiaqi Yu, Lishuai Feng, Yunpeng Tang, Nianhui Yu, Jianning Lin, Yuan Ji, Hui Li

**Affiliations:** 1 Department of Radiology, Shanghai Jiao Tong University School of Medicine Affiliated Shanghai Sixth People’s Hospital, 600 Yi Shan Road, Shanghai 200233, China; yujiaqi1997@alumni.sjtu.edu.cn (J.Y.); fls1991@sjtu.edu.cn (L.F.); maya_xka@sjtu.edu.cn (Y.T.); ynh19971007@alumni.sjtu.edu.cn (N.Y.); linjn2220@sjtu.edu.cn (J.L.); 2 Department of Pathology, Zhongshan Hospital, Fudan University, Shanghai 200230, China

**Keywords:** pancreatic cancer, chemodynamic therapy, manganese dioxide, hydrogen peroxide, autophagy

## Abstract

**Background/Objectives:** Conventional therapeutic strategies exhibit limited efficacy against pancreatic cancer, primarily due to its profoundly hypoxic tumor microenvironment and dense fibrotic stroma. Chemodynamic therapy (CDT) holds promise; however, its application in pancreatic cancer is restricted by insufficient endogenous hydrogen peroxide (H_2_O_2_) levels and the activation of protective autophagy in response to oxidative stress. **Methods:** To overcome these obstacles, we developed a tumor microenvironment-responsive, pancreatic cancer-targeted CDT nanoamplifier—H-MnO_2_/GOX&CQ-iRGD—comprising a hollow mesoporous MnO_2_ shell co-loaded with glucose oxidase (GOX) and chloroquine (CQ), and surface-functionalized with the tumor-penetrating peptide iRGD. GOX catalyzes glucose oxidation to generate H_2_O_2_, enhancing Fenton-like reactions. CQ suppresses autophagy induced by oxidative stress, thereby alleviating therapy resistance. The iRGD peptide targets integrin α_v_β_3_, which is overexpressed on pancreatic cancer cells and tumor vasculature, promoting deep tumor penetration and enhanced delivery efficiency. **Results:** We comprehensively characterized the nanoplatform’s physicochemical properties, tumor microenvironment triggered degradation, controlled drug release, glucose-driven H_2_O_2_ generation, and hydroxyl radical production in vitro. Cellular studies assessed nanoparticle uptake, intracellular H_2_O_2_ production, autophagy inhibition, and cytotoxicity. In vivo experiments further demonstrated effective tumor targeting and significant therapeutic outcomes in pancreatic cancer models. **Conclusions:** This nanoplatform addresses major barriers of CDT—namely, insufficient H_2_O_2_ levels, autophagy-mediated resistance, and limited intratumoral penetration—offering a promising strategy for pancreatic cancer treatment.

## 1. Introduction

Pancreatic cancer is one of the most aggressive and lethal malignancies, characterized by an insidious onset, rapid progression, and extremely poor prognosis, with a 5-year survival rate of less than 5% [1]. A hallmark of pancreatic tumors is persistent and profound hypoxia, resulting from inadequate vascularization. This hypoxic tumor microenvironment (TME) plays a critical role in tumor initiation, progression, metastasis, and resistance to therapy. In addition, the dense fibrotic stromal matrix within pancreatic tumors forms a formidable physical barrier that severely restricts the penetration and efficacy of therapeutic agents. These unique pathological features highlight the urgent need for innovative and effective treatment strategies for pancreatic cancer [2].

Chemodynamic therapy (CDT) has emerged as a promising strategy, leveraging hallmark features of the TME, such as acidic pH, hypoxia, abnormal vasculature, and elevated levels of hydrogen peroxide (H_2_O_2_) and glutathione (GSH) [3,4]. CDT utilizes nanodrugs capable of generating cytotoxic hydroxyl radicals (∙OH) through Fenton or Fenton-like reactions within the TME, thereby inducing tumor-specific oxidative damage. Compared with traditional treatments such as chemotherapy and radiotherapy, CDT offers distinct advantages including higher specificity, minimal systemic toxicity, deeper tissue penetration, and the ability to circumvent multidrug resistance mechanisms [5,6,7,8,9]. However, the application of CDT in pancreatic cancer faces three major challenges: (i) insufficient intratumoral H_2_O_2_ and excessive GSH-mediated ·OH clearance [10,11,12,13]; (ii) protective autophagy triggered by oxidative stress leads to therapeutic resistance [14,15]; (iii) the dense fibrotic stroma that significantly impedes the delivery of therapeutic agents and nanoparticles [16,17]. Therefore, the development of a targeted multifunctional platform that can simultaneously generate H_2_O_2_ in situ, deplete intracellular GSH, and inhibit autophagy is urgently needed to improve CDT efficacy in pancreatic cancer.

**Scheme 1:**
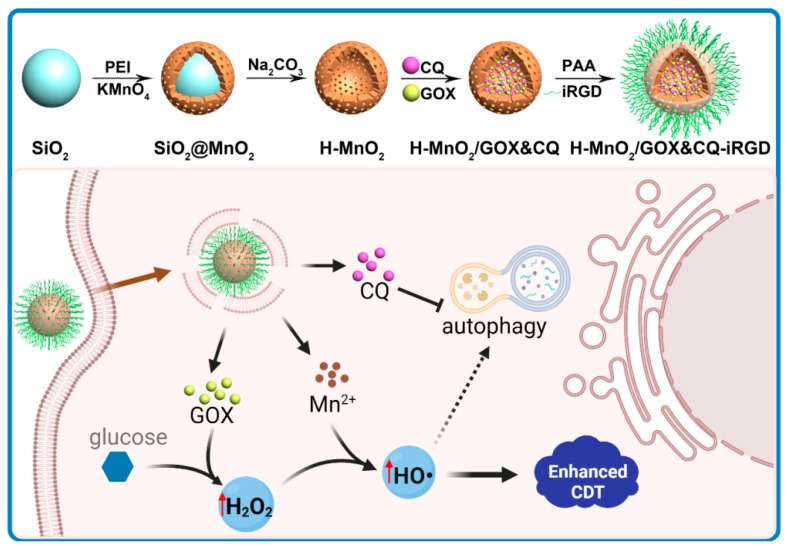
Schematic illustration of the construction of H-MnO_2_/GOX&CQ-iRGD and its mechanism of action in pancreatic tumor chemodynamic therapy. The iRGD peptide mediates nanoparticle penetration through the dense stromal barrier. In the acidic TME, sequential degradation releases Mn^2+^, O_2_, GOX, and CQ. GOX catalyzes glucose oxidation to generate H_2_O_2_, which reacts with Mn^2+^ via Fenton chemistry to produce hydroxyl radicals (∙OH), to enhance the chemodynamic effect and induce tumor cell death. Concurrently, oxidative damage may trigger protective autophagy in tumor cells, which is effectively suppressed by CQ through inhibition of autolysosomal degradation, thereby reducing tumor cell resistance to CDT and further amplifying the therapeutic efficacy.

Herein, we designed a cascade nanoamplifier-targeting pancreatic cancer to enhance the therapeutic efficacy of CDT. This multifunctional nanoamplifier, termed H-MnO_2_/GOX&CQ-iRGD, integrates four synergistic components (Scheme 1). First, hollow manganese dioxide (H-MnO_2_) degrades in the acidic TME, releasing Mn^2+^ and O_2_, which generate cytotoxic ∙OH and alleviate hypoxia, while also depleting intracellular GSH, thereby weakening the tumor’s antioxidant defense system [18,19,20]. Second, GOX catalyzes the oxidation of glucose to produce H_2_O_2_, further amplifying ∙OH production and enhancing Fenton-like reactions. Specifically, MnO_2_ decomposes in the acidic tumor microenvironment to generate Mn^2+^ ions, which catalyze the conversion of endogenous H_2_O_2_ into highly reactive ∙OH via a Fenton-like reaction. These radicals cause oxidative damage to cellular components, leading to apoptosis or necrosis. This mechanism underlies the core of CDT and is further enhanced in our system by GOX-mediated self-supply of H_2_O_2_ [21,22,23]. Third, CQ acts as an autophagy inhibitor by accumulating in acidic organelles such as lysosomes, where it disrupts acidification and blocks autophagosome–lysosome fusion, thereby inhibiting autophagic flux. This enhances oxidative stress, sensitizes tumor cells to oxidative damage, and helps prevent the development of therapeutic resistance [24,25,26,27]. To overcome the dense stromal barrier in pancreatic cancer, we functionalized the nanoparticle surface with iRGD, a tumor-penetrating peptide that targets integrin α_v_β_3_—overexpressed on both pancreatic cancer cells and tumor vasculature—thereby promoting deep tumor penetration [28,29,30,31,32].

To initiate the cascade, the H-MnO_2_ nanoparticles were synthesized using polyethyleneimine (PEI) as a reducing agent, followed by the loading of GOX and CQ and the surface conjugation of iRGD. This nanoplatform degrades rapidly in the TME, ensuring timely and localized drug release. Compared to free drugs, H-MnO_2_/GOX&CQ-iRGD exhibits superior tumor penetration, targeting ability, reactive oxygen species (ROS) generation, and autophagy inhibition. In vivo tumor suppression studies further support its efficacy: through a sequential “targeting–dissociation–catalysis–inhibition” cascade, this nanoamplifier effectively addresses the major limitations of CDT, offering a promising strategy for enhancing CDT outcomes in pancreatic cancer.

## 2. Materials and Methods

### 2.1. Materials

Chloroquine (CQ) was purchased from Sigma-Aldrich (St. Louis, MO, USA). The bifunctional cyclic peptide iRGD was obtained from MedChemExpress (Monmouth Junction, NJ, USA). Glucose (Glu), glucose oxidase (GOX), 2,2′-azino-bis(3-ethylbenzothiazoline-6-sulfonic acid) diammonium salt (ABTS), horse-radish peroxidase (HRP), and methylene blue (MB) were all sourced from Sigma-Aldrich (St. Louis, MO, USA), unless otherwise stated. Additionally, methylene blue (MB) was supplied by Shanghai Macklin Biochemical Co., Ltd. (Shanghai, China). Cell culture medium and fetal bovine serum (FBS) were procured from Gibco (Waltham, MA, USA), whereas trypsin and penicillin–streptomycin solution were acquired from Yeasen Bio-technology Co., Ltd. (Shanghai, China). Dihydroethidium (DHE) and p62 antibody were purchased from Beyotime Biotechnology (Shanghai, China). High-glucose Dulbecco’s Modified Eagle Medium (DMEM) base medium was obtained from Hyclone (Logan, UT, USA). Fluorescent labeling dyes Cy7 and Cy5 were obtained from Bioss (Beijing, China). Human pancreatic cancer cell line (PANC-1) was obtained from the Cell Bank of the Chinese Academy of Sciences (Shanghai, China). Female Balb/c nude mice were purchased from Shanghai SLAC Laboratory Animal Co., Ltd. (Shanghai, China).

Transmission electron microscope (Tecnai G2 20, ThermoFisher Scientific, Waltham, MA, USA) and field emission transmission electron microscope (Tecnai G2 F20 S-Twin, ThermoFisher Scientific, Waltham, MA, USA) were employed for internal structural characterization, while a field emission scanning electron microscope (Ultra 55, Carl Zeiss, Oberkochen, Germany) was used to observe surface morphology. Fourier-transform infrared spectrometer (Nicolet 6700, ThermoFisher Scientific, Waltham, MA, USA), X-ray diffractometer (Bruker D8, Bruker, Billerica, MA, USA), ultraviolet–visible spectrophotometer (UV-2600, Shimadzu, Kyoto, Japan), X-ray photoelectron spectrometer (PHI 5300, Physical Electronics, Chanhassen, MN, USA), and inductively coupled plasma optical emission spectrometer (iCAP 7400, ThermoFisher Scientific, Waltham, MA, USA) were used for physicochemical analysis. A nanoparticle size and zeta potential analyzer (Zetasizer Nano ZS90, Malvern Panalytical, Malvern, Worcestershire, UK) and an automated surface area and porosity analyzer (AUTOSORB-IQ, Quantachrome Instruments [now Anton Paar QuantaTec], Boynton Beach, FL, USA) were applied to evaluate colloidal stability and pore structures. A headspace residual oxygen analyzer (OXY-1STDZ, PreSens, Regensburg, Germany) was used to monitor oxygen generation. Confocal laser scanning microscope (Olympus, Tokyo, Japan) and inverted microscope (Nikon, Tokyo, Japan) were employed for cellular imaging, while a Western blot electrophoresis system (Bio-Rad, Hercules, CA, USA) and protein band imaging system (Bio-Rad, Hercules, CA, USA) were used for protein expression analysis. A small animal in vivo imaging system (LB983, Berthold Technologies, Bad Wildbad, Germany) was utilized to monitor nanoparticle biodistribution in mice. Additional supporting equipment included an ultrasonic cleaner (Ningbo Qingzhi Biotechnology Co., Ltd., Ningbo, China), a high-speed centrifuge (Xiangyi, Hunan, China), a freeze dryer (Ningbo Qingzhi Biotechnology Co., Ltd., Ningbo, China), a thermostatic water bath (HWS-12, Yiheng, Shanghai, China), an ultraclean workbench (Shanghai Yuejin Medical Equipment Co., Ltd., Shanghai, China), and a single-channel syringe pump (LSP01-2A, Longer Pump, Baoding, China).

### 2.2. Methodology

#### 2.2.1. Construction and Characterization of H-MnO_2_/GOX&CQ-iRGD

Initially, SiO_2_ nanoparticles were synthesized. In a 200 mL round-bottom flask, 60 mL of absolute ethanol, 2 mL of deionized water, and 2.4 mL of ammonia solution were added and stirred for 10 min. A mixture of 5.9 mL of tetraethyl orthosilicate (TEOS) and 15 mL of absolute ethanol was then prepared and injected into the above solution at a rate of 10 mL/h using a syringe pump. The mixture was continuously stirred at 300 rpm at room temperature for 12 h. The resulting product was collected by centrifugation and washed repeatedly with absolute ethanol and deionized water, then dispersed in 50 mL of deionized water to obtain SiO_2_ nanoparticles.

Next, the as-prepared SiO_2_ nanoparticles were mixed with 2 mL of branched PEI aqueous solution (10 mg/mL) in a 200 mL round-bottom flask and stirred for 2 h. The product was collected by centrifugation at 13,000 rpm for 10 min and washed three times with deionized water, then dispersed in 50 mL of water. Subsequently, 10 mL of KMnO_4_ aqueous solution (20 mg/mL) was added and the mixture was shaken for 6 h. After centrifugation at 13,000 rpm for 10 min and washing three times with deionized water, SiO_2_@MnO_2_ core–shell nanoparticles were obtained.

The product was then dispersed in mL of 1 M Na_2_CO_3_ solution and reacted at 60 °C for 12 h to etch the silica core. After the reaction, the product was centrifuged at 13,000 rpm for 10 min, washed three times with deionized water, and finally dispersed in 50 mL of deionized water to obtain hollow MnO_2_ nanoparticles (H-MnO_2_) for subsequent experiments [33,34].

The internal structure and elemental distribution of H-MnO_2_ were characterized using transmission electron microscopy (TEM) and high-resolution TEM (HRTEM). The surface morphology was examined by field-emission scanning electron microscopy (FESEM). The nitrogen adsorption–desorption isotherms and pore size distributions were analyzed using an automated surface area and porosity analyzer.

SiO_2_, SiO_2_@PEI, SiO_2_@MnO_2_, and H-MnO_2_ at a concentration of 1 mg/mL were dispersed in deionized water, and their hydrodynamic diameters and zeta potentials were measured using a nanoparticle size and zeta potential analyzer. The lyophilized H-MnO_2_ powder was analyzed by X-ray photoelectron spectroscopy (XPS) to determine its composition and valence states, as well as by an automated surface area and porosity analyzer to evaluate its specific surface area and pore size distribution.

Different concentrations of GOX (750 μg/mL) and CQ (500 μg/mL) in DMSO were added to 20 mL of H-MnO_2_ solution (5 mg/mL) and stirred for 24 h in the dark. Subsequently, 5 mL of PEI aqueous solution (10 mg/mL) was added and stirred for an additional hour. After the reaction, the product was centrifuged at 13,000 rpm for 10 min, washed three times with deionized water, and dispersed in 20 mL of deionized water to obtain H-MnO_2_/GOX&CQ@PEI nanoparticles. To remove unbound CQ and GOX, the nanoparticles were purified by repeated centrifugation (13,000 rpm, 10 min) and triple washing with deionized water. This method utilizes the size and solubility differences between the nanoparticles and small-molecule drugs: the larger nanoparticles sediment under high-speed centrifugation, while free CQ and GOX remain in the supernatant and are discarded during washing. This purification approach is widely used in nanoparticle preparation to eliminate free drug residues.

Next, 5 mL of polyacrylic acid (PAA) aqueous solution (10 mg/mL) was added to 10 mL of H-MnO_2_/GOX&CQ@PEI solution (10 mg/mL) and stirred for 2 h. After centrifugation at 13,000 rpm for 10 min and washing three times with deionized water, the resulting H-MnO_2_/GOX&CQ@PAA nanoparticles were dispersed in 10 mL of deionized water.

Then, 5 mg of 1-ethyl-3-(3-dimethylaminopropyl)carbodiimide (EDC) and 3.5 mg of 4-dimethylaminopyridine (DMAP) were added, and the mixture was reacted in the dark at 300 rpm for 2 h to activate the carboxyl groups. After centrifugation at 13,000 rpm for 10 min, 10 mL of iRGD solution (1 mg/mL) was added and stirred in the dark for 24 h. The product was collected by centrifugation at 13,000 rpm for 10 min and washed three times with deionized water to obtain H-MnO_2_/GOX&CQ-iRGD [35,36,37].

Finally, the synthesized manganese-based CDT amplifier, H-MnO_2_/GOX&CQ-iRGD, was systematically characterized using Fourier-transform infrared spectroscopy (FTIR), UV–Vis spectroscopy, and a nanoparticle size and zeta potential analyzer.

#### 2.2.2. Detection of O_2_, H_2_O_2_, and ·OH Generation and the Release of Mn^2+^, CQ, and GOX from H-MnO_2_/GOX&CQ-iRGD Nanoparticles

The oxygen-generating capacity of H-MnO_2_/GOX&CQ-iRGD was evaluated using a headspace residual oxygen analyzer in 20 mL glass vials. Specifically, 10 mL of a simulated TME solution (pH 6.5 containing H_2_O_2_ and GSH; GSH: 5 mM, H_2_O_2_: 100 μM) was added into each vial. The solution was purged with nitrogen for 30 min to remove dissolved oxygen. An oxygen sensor was then inserted into the sealed vial, and different concentrations of H-MnO_2_/GOX&CQ-iRGD were injected. The change in O_2_ concentration was monitored over time.

For H_2_O_2_ generation analysis, four groups were prepared: Control (deionized water), H-MnO_2_ group (100 μM Mn^2+^), GOX group (10 μg/mL GOX), and H-MnO_2_/GOX group (100 μM Mn^2+^ + 10 μg/mL GOX). In each group, 1 mL of sample solution was mixed with 1 mL of glucose solution (10 μg/mL) and 10 μL of ABTS solution (5 mM), followed by incubation at 37 °C in the dark for 1 h. The absorbance of ABTS was then measured using a UV–Vis spectrophotometer in the range of 470–800 nm.

For ·OH generation analysis, 1 mL of each group solution was mixed with 1 mL of glucose solution (10 μg/mL), 25 μL of NaHCO_3_ solution (1 M), and 10 μL of methylene blue (MB) solution (5 mM). The mixture was incubated at 37 °C in the dark for 1 h, and the absorbance of MB at 664 nm was measured using a UV–Vis spectrophotometer.

The release profiles of Mn^2+^, CQ, and GOX were investigated by placing 10 mg of H-MnO_2_/GOX&CQ-iRGD into a dialysis bag, which was then immersed in 100 mL of different solutions: pH 7.4, pH 6.5, pH 6.5 + H_2_O_2_, and pH 6.5 + H_2_O_2_ + GSH (GSH: 5 mM, H_2_O_2_: 100 μM). The solutions were oscillated at 37 °C and 200 rpm. At predetermined time points (0, 5, 10, 15, 20, 30, 40, 50, and 60 min), 2 mL of release medium was collected and replaced with an equal volume of fresh medium. The concentration of Mn^2+^ was measured using inductively coupled plasma optical emission spectrometry (ICP-OES), while the concentrations of CQ and GOX were determined by UV–Vis spectrophotometry.

#### 2.2.3. Therapeutic Study of H-MnO_2_/GOX&CQ-iRGD on Human Pancreatic Cancer Cells

The cytotoxicity of H-MnO_2_/GOX&CQ-iRGD was first evaluated in vitro. Human pancreatic cancer cells (PANC-1) were cultured in Dulbecco’s Modified Eagle Medium (DMEM) supplemented with 10% fetal bovine serum (FBS) and 1% penicillin–streptomycin, and incubated in a humidified atmosphere at 37 °C with 5% CO_2_ for 24 h.

PANC-1 cells were then seeded into 96-well plates at a density of 1 × 10^5^ cells per well and cultured for an additional 24 h. Subsequently, different concentrations of H-MnO_2_-iRGD, H-MnO_2_/CQ-iRGD, H-MnO_2_/GOX-iRGD, and H-MnO_2_/GOX&CQ-iRGD were added to the wells in DMEM medium and incubated for another 24 h. The concentrations used were as follows: H-MnO_2_ at 100, 50, 25, 12.5, and 6.25 μg/mL; GOX at 10, 5, 2.5, 1.25, and 0.63 μg/mL; and CQ at 15, 7.5, 3.75, 1.88, and 0.94 μg/mL.

After incubation, the medium was removed, and 100 μL of fresh medium and 100 μL of MTT solution (1 mg/mL) were added to each well. The cells were incubated for another 4 h. The absorbance at 490 nm was measured using a microplate reader to quantify MTT reduction, reflecting cellular metabolic activity.

Additionally, to evaluate the tumoricidal activity of H-MnO_2_/GOX&CQ-iRGD against pancreatic cancer cells, PANC-1 cells were treated with appropriate concentrations of H-MnO_2_-iRGD (100 μg/mL), H-MnO_2_/CQ-iRGD (CQ: 15 μg/mL), H-MnO_2_/GOX-iRGD (GOX: 10 μg/mL), and H-MnO_2_/GOX&CQ-iRGD (H-MnO_2_: 100 μg/mL; GOX: 10 μg/mL; CQ: 15 μg/mL) for 24 h. After treatment, relative metabolic activity was determined using a CCK-8 assay.

In 10 mL of H-MnO_2_/GOX&CQ@PEI solution (10 mg/mL), 1 mL of Cy5 solution (1 mg/mL), 2 mg of EDC, and 1.4 mg of DMAP were added. The mixture was stirred at 300 rpm under dark conditions for 24 h. After the reaction, the product was collected by centrifugation at 13,000 rpm for 10 min and washed three times with deionized water to obtain Cy5-H-MnO_2_/GOX&CQ@PEI, which was then dispersed in 10 mL of deionized water. Next, 5 mL of PAA aqueous solution (10 mg/mL) was added and stirred for 2 h. The product was again collected by centrifugation at 13,000 rpm for 10 min, washed three times with deionized water, and dispersed in 10 mL of deionized water to obtain Cy5-H-MnO_2_/GOX&CQ@PAA. Subsequently, 5 mg of EDC and 3.5 mg of DMAP were added to the dispersion and reacted at 300 rpm under dark conditions for 2 h to activate the carboxyl groups. After centrifugation at 13,000 rpm for 10 min, 10 mL of iRGD solution (1 mg/mL) was added and stirred under dark conditions for 24 h. The final product was collected by centrifugation at 13,000 rpm for 10 min, washed three times with deionized water, and dispersed in 10 mL of deionized water to obtain Cy5-H-MnO_2_/GOX&CQ-iRGD. Subsequently, we fabricated Cy7-H-MnO_2_/GOX&CQ-iRGD employing the identical procedure. This final Cy7-labeled nanoplatform was used for in vivo imaging of the CDT amplifier.

PANC-1 cells were seeded into confocal culture dishes at a density of 3 × 10^5^ cells per well. After 24 h of incubation, the cells were treated with Cy5-labeled H-MnO_2_/GOX&CQ-iRGD (10 μg/mL) for 1, 2, and 3 h, respectively. Following incubation, the cells were washed with PBS and then fixed with 1 mL of 2.5% glutaraldehyde for 10 min. Finally, the cellular internalization of the nanoplatform was visualized and imaged using a confocal laser scanning microscope.

PANC-1 cells were seeded into 24-well plates at a density of 5 × 10^4^ cells per well. After 24 h of incubation, the cells were treated with different formulations, including Control, H-MnO_2_-iRGD, H-MnO_2_/GOX-iRGD, H-MnO_2_/CQ-iRGD, and H-MnO_2_/GOX&CQ-iRGD, for 6 h. Following treatment, the cells were fixed with 4% paraformaldehyde and 0.2% Triton X-100 for 15 min. Subsequently, they were incubated with the p62 antibody at room temperature for 2 h. The cells were then washed three times with PBS and incubated with FITC-labeled IgG in the dark for 5 h. Autophagosomes were visualized and recorded using a confocal laser scanning microscope.

For intracellular ROS detection, a separate set of fixed cells from each group was incubated with DHE for 10 min, followed by washing three times with PBS. The cells were then imaged using confocal microscopy to assess intracellular ROS levels.

For total cellular protein extraction, 200 μL of radioimmunoprecipitation assay (RIPA) lysis buffer was added to each tube of treated cells, followed by pipetting approximately 200 times to ensure thorough lysis. The lysates were centrifuged at 12,000 rpm for 10 min at 4 °C. The supernatants were collected and stored at –80 °C for subsequent Western Blot analysis.

#### 2.2.4. Imaging and Therapeutic Evaluation of H-MnO_2_/GOX&CQ-iRGD in Pancreatic Tumor-Bearing Mice

Healthy female nude mice (6 weeks old) were intravenously injected via the tail vein with H-MnO_2_/GOX&CQ-iRGD at a dose of 20 mg/kg to evaluate the biocompatibility of the nanoplatform. On days 1 and 3 post-injection, the mice were sacrificed for blood collection and organ harvesting. Hematological parameters, including mean corpuscular volume (MCV), mean corpuscular hemoglobin (MCH), mean corpuscular hemoglobin concentration (MCHC), mean platelet volume (MPV), platelet count (PLT), hematocrit (HCT), hemoglobin (HGB), red blood cell count (RBC), red cell distribution width–coefficient of variation (RDW-CV), and white blood cell count (WBC), were analyzed in parallel among three groups to assess systemic toxicity. Major organs, including the heart, liver, spleen, lungs, and kidneys, were collected and subjected to hematoxylin and eosin (H&E) staining for histological evaluation.

To evaluate therapeutic efficacy, we employed a subcutaneous PANC-1 pancreatic tumor model by injecting PANC-1 cells into the right forelimb of BALB/c nude mice. For in vivo imaging, 100 μL of H-MnO_2_/GOX&CQ-iRGD solution labeled with Cy7 fluorescent dye (10 mg/mL) was intravenously injected via the tail vein. Small animal fluorescence imaging (LB983, Berthold Technologies) was performed at 0, 3, 6, 12, 18, and 24 h post-injection to evaluate the biodistribution and tumor accumulation of H-MnO_2_/GOX&CQ-iRGD in the pancreatic tumor region.

Once tumors reached approximately 100 mm^3^, the mice were randomly assigned to five treatment groups (*n* = 5 per group): (1) saline control, (2) H-MnO_2_-iRGD, (3) H-MnO_2_/GOX-iRGD, (4) H-MnO_2_/CQ-iRGD, and (5) H-MnO_2_/GOX&CQ-iRGD. Treatments were administered intravenously once per week for four consecutive weeks. Tumor volume, body weight, and overall survival were monitored every four days and recorded photographically.

At the end of four weeks, the surviving mice were sacrificed. Major organs, including the heart, liver, spleen, lungs, kidneys, as well as the tumors, were harvested. The collected tissues were subjected to H&E staining, terminal deoxynucleotidyl transferase dUTP nick end labeling (TUNEL) staining, and immunohistochemical analysis of CD68, MCP-1, MPO, and TGF-β in pancreatic tumor tissues.

For all quantitative experiments, unless otherwise specified, each experiment was independently repeated three times (*n* = 3).

### 2.3. Statistical Analysis

All experiments were conducted independently at least three times to ensure reproducibility and reliability. Data were analyzed using GraphPad Prism software version 9.0 (GraphPad Software, Inc., San Diego, CA, USA). Statistical analysis was performed using one-way or two-way ANOVA, followed by appropriate multiple comparison tests, depending on the experimental design. The significance level is set as the probability of * *p* < 0.05, ** *p* < 0.01, *** *p* < 0.001, and **** *p* < 0.0001.

## 3. Results

### 3.1. Construction and Characterization of the Cascade Nanoamplifier

The cascade nanoamplifier was synthesized by first preparing monodisperse SiO_2_ nanoparticles via a controlled sol–gel method, which served as sacrificial templates. MnO_2_ was then deposited onto the SiO_2_ cores through a BPEI-mediated redox reaction with KMnO_4_, forming SiO_2_@MnO_2_ core–shell structures. Selective etching of the silica cores produced mesoporous H-MnO_2_ nanospheres. The morphological evolution during synthesis is illustrated in Figure S1A–D. TEM images confirmed uniform particle size and good monodispersity (Figure S1E–G). The particle sizes of SiO_2_, SiO_2_@MnO_2_, and H-MnO_2_ were measured to be 115, 136, and 138 nm, respectively (Figure S1H). The zeta potential results showed that the surface charges of SiO_2_, SiO_2_@PEI, SiO_2_@MnO_2_, and H-MnO_2_ were –28.8, 34.3, –24.6, and –20.3 mV, respectively, which aligns well with the designed synthetic strategy (Figure S1I). SEM images further revealed that the synthesized H-MnO_2_ possesses a porous surface structure while maintaining structural integrity (Figure S1J).Elemental mapping indicated homogeneous distribution of Mn and O (Figure S1K), and X-ray photoelectron spectroscopy revealed a Mn:O atomic ratio of approximately 1:2.9, with the excess oxygen likely originating from adsorbed species such as CO_2_ (Figure S1L). High-resolution spectra showed characteristic Mn 2p peaks at 654.0 eV (2p_1_/_2_) and 642.8 eV (2p_3_/_2_), consistent with Mn(IV), and an energy separation of Δ = 11.2 eV (Figure S1M). XRD patterns indicated an amorphous structure, which is generally associated with higher catalytic reactivity than crystalline forms (Figure S1N). These results confirm the successful synthesis of structurally intact, mesoporous H-MnO_2_ nanospheres with uniform size, good monodispersity and amorphous structure.

**Figure 1:**
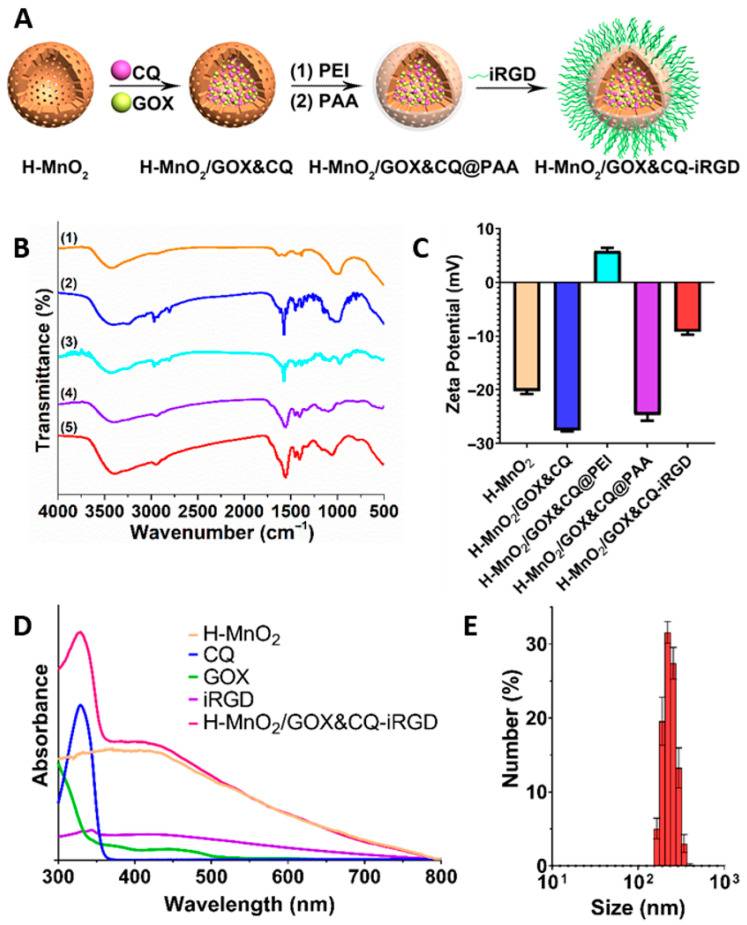
Construction and physicochemical characterization of H-MnO_2_/GOX&CQ-iRGD. (**A**) Stepwise synthetic route: silica templating → PEI-mediated MnO_2_ shell formation → core removal → sequential loading of GOX and CQ → PEI and PAA surface coatings → iRGD conjugation. (**B**) FTIR spectra of intermediates: (1) H-MnO_2_, (2) H-MnO_2_/GOX&CQ, (3) H-MnO_2_/GOX&CQ@PEI, (4) H-MnO_2_/GOX&CQ@PAA, and (5) H-MnO_2_/GOX&CQ-iRGD. (**C**) Zeta potentials of samples 1–5, illustrating surface charge shifts following each modification step. (**D**) UV–Vis absorption spectra of H-MnO_2_ (5 mg mL^−1^), CQ (500 μg mL^−1^), GOX (750 μg mL^−1^), iRGD (1 mg mL^−1^), and the final H-MnO_2_/GOX&CQ-iRGD, showing characteristic peaks at 329 nm (CQ) and 445 nm (GOX). (**E**) Hydrodynamic diameter distribution of H-MnO_2_/GOX&CQ-iRGD measured by DLS, centered at ~200 nm with narrow polydispersity.

In subsequent steps, the multifunctional H-MnO_2_/GOX&CQ-iRGD nanoamplifier was constructed. To minimize steric hindrance, GOX was loaded into the hollow cavities prior to CQ. To prevent premature drug leakage, the surface was sealed with a PEI layer, then coated with PAA to improve colloidal stability and reduce nonspecific protein adsorption. Finally, the tumor-penetrating peptide iRGD was conjugated to achieve targeted delivery, completing the TME-responsive nanoplatform for enhanced chemodynamic therapy (Figure 1A). Following the successful synthesis of the nanoamplifier, the potential for drug loading and delivery was evaluated by characterizing their nitrogen (N_2_) adsorption-desorption isotherms and pore size distribution using an automated specific surface area and porosity analyzer. As shown in Figure S2A,B, the isotherms correspond to a typical type IV profile with a distinct hysteresis loop during desorption, indicating the presence of abundant mesoporous structures.(pore size: 5.62 nm; specific surface area: 155.97 m^2^/g). These results suggest that the hollow mesoporous architecture of H-MnO_2_ provides an excellent platform for drug loading, enabling high drug payloads for potential application in pancreatic cancer therapy.

Comprehensive characterization confirmed successful assembly of the nanoplatform. FTIR spectra exhibited clear changes in surface functional groups at each stage of modification (Figure 1B). Correspondingly, zeta potential measurements showed stepwise shifts from −20.27 mV (H-MnO_2_), to −27.67 mV (GOX&CQ-loaded), +5.87 mV (PEI-coated), −24.77 mV (PAA-coated), and −9.23 mV (iRGD-modified) (Figure 1C), confirming each modification layer. UV–Vis spectroscopy revealed characteristic absorption peaks at 445 nm (GOX) and 329 nm (CQ), demonstrating successful drug encapsulation (Figure 1D and Figure S2C–F). Quantitative analysis indicated a final mass ratio of H-MnO_2_:GOX:CQ ≈ 10:1:1.5. The loading content of CQ and GOX were determined to be 12 wt% and 8 wt%, the encapsulation efficiencies of GOX and CQ in H-MnO2/GOX nanoparticles are approximately 75.2% and 70.6%, respectively [38]. DLS analysis showed a hydrodynamic diameter of ~200 nm with uniform size distribution and good colloidal stability, indicating excellent integrity and suitability for biological applications (Figure 1E).

### 3.2. TME-Induced Shell Collapse and Enhanced Fenton Reaction of the Cascade Nanoamplifier (Solution Level)

**Figure 2:**
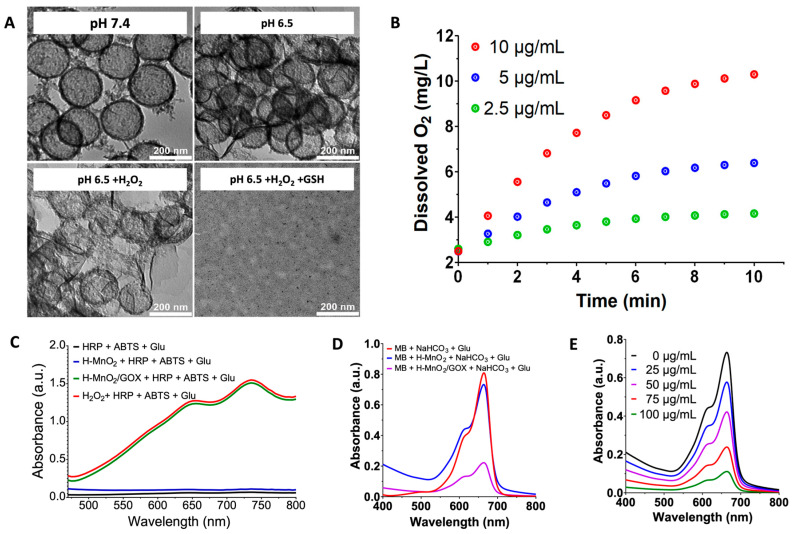
Solution-level evaluation of H-MnO_2_/GOX&CQ-iRGD in vitro. (**A**) TEM images after 12 h incubation under: pH 7.4; pH 6.5; pH 6.5 + 100 μM H_2_O_2_; pH 6.5 + 100 μM H_2_O_2_ + 5 mM GSH, illustrating pH/GSH-triggered shell collapse. (**B**) O_2_ concentration over time at different concentrations of H-MnO_2_/GOX&CQ-iRGD in simulated TME solution (hypoxia, pH 6.5 + H_2_O_2_ + GSH, GSH: 5 mM, H_2_O_2_: 100 μM). (**C**) Time-dependent H_2_O_2_ generation in tumor-mimicking buffer (pH 6.5 + H_2_O_2_ + GSH), measured by HRP–ABTS assay. (**D**) UV–Vis spectra of MB after 2 h treatment under different conditions, indicating ∙OH-mediated MB degradation. (**E**) MB degradation following 2 h incubation with increasing concentrations of H-MnO_2_/GOX and glucose, showing a dose-dependent ∙OH generation.

To investigate the TME-responsiveness of the system, we simulated the pancreatic TME (pH 6.5, ~100 μM H_2_O_2_, ~5 mM GSH) [39,40,41] and monitored degradation and drug release behavior. H-MnO_2_/GOX&CQ-iRGD remained stable at pH 7.4, the high structural stability of H-MnO_2_/GOX&CQ-iRGD under physiological conditions minimizes damage to normal tissues/organs during blood circulation. Slight degradation occurred under mildly acidic conditions (pH 6.5), while complete collapse of the MnO_2_ shell was observed in the presence of both H_2_O_2_ and GSH at pH 6.5, mimicking pancreatic TME conditions (Figure 2A).

Based on these findings, we further evaluated the release behavior of Mn^2+^, GOX, and CQ from H-MnO_2_/GOX&CQ-iRGD using ICP-OES and UV–Vis spectroscopy under various conditions (pH 7.4, pH 6.5, pH 6.5 + H_2_O_2_, and pH 6.5 + H_2_O_2_ + GSH). As shown in Figure S3A, the cumulative release percentage of Mn^2+^ under different conditions further illustrates the degradation process of the MnO_2_ shell. For instance, after 30 min of reaction, the Mn^2+^ release rates in pH 7.4, pH 6.5, pH 6.5 + H_2_O_2_, and pH 6.5 + H_2_O_2_ + GSH were 4.3%, 13.8%, 24.6%, and 95.8%, respectively, which is consistent with Figure 2A. Along with the degradation of the MnO_2_ shell, the loaded drugs GOX and CQ were released, with release trends closely matching that of MnO_2_ degradation. For example, after 30 min, the release rates of GOX under the same conditions were 1.3%, 8.5%, 21.7%, and 87.4%, respectively, while the corresponding CQ release rates were 2.8%, 8.9%, 23.9%, and 85.4%. These results indicate that the drug release is a direct consequence of the degradation of the MnO_2_ shell (Figure S3B,C). These results confirm that the nanoplatform is stable under physiological conditions but rapidly degrades under tumor-specific stimuli, enabling selective and efficient release of GOX and CQ within the tumor site.

To address the TME-hypoxic limitation of GOX-mediated H_2_O_2_ generation, we next assessed the O_2_-generating capacity of H-MnO_2_/GOX&CQ-iRGD under pancreatic TME-like conditions. H-MnO_2_/GOX&CQ-iRGD exhibited sustained, concentration-dependent O_2_ release (Figure 2B), effectively alleviating local hypoxia and thus enhancing GOX-catalyzed H_2_O_2_ production. This O_2_ supplementation contributes significantly to improving the CDT efficacy of the nanoplatform under tumor-mimicking conditions.

To assess the catalytic performance of the cascade nanoamplifier in generating H_2_O_2_, a colorimetric assay based on horseradish peroxidase (HRP)-mediated ABTS oxidation was conducted. This method detects H_2_O_2_ generated from the enzymatic reaction between glucose and the glucose oxidase (GOX)-loaded nanoparticle. As shown in Figure 2C, H-MnO_2_ alone failed to trigger ABTS oxidation, indicating that it lacks intrinsic catalytic activity for glucose oxidation. In contrast, the addition of GOX-loaded H-MnO_2_/GOX resulted in a significant increase in absorbance within the 470–800 nm range, comparable to the response observed with exogenous H_2_O_2_. These results confirm that H-MnO_2_/GOX effectively catalyzes glucose oxidation to generate H_2_O_2_.

Building on this finding, we further investigated the system’s ability to produce hydroxyl radicals (∙OH) under simulated TME conditions. Methylene blue (MB), a classical ∙OH-sensitive probe, was used to monitor ∙OH generation. Hydroxylation of MB by ∙OH leads to a decrease in its characteristic absorbance at 664 nm. As shown in Figure 2D, treatment with H-MnO_2_/GOX resulted in an approximately 75% reduction in MB absorbance, indicating substantial ∙OH production via the Fenton-like reaction. Moreover, a concentration-dependent decrease in absorbance at 664 nm was observed with increasing concentrations of H-MnO_2_/GOX (Figure 2E), further supporting the efficient and scalable ∙OH generation capability of the system.

Collectively, these results demonstrated that the cascade nanoamplifier can effectively disintegrate in the pancreatic TME and significantly enhance the Fenton reaction at the solution-level. These results inspired to further study of its performance at the cellular-level.

### 3.3. Pancreatic Cancer Cell-Specific Cascade Nanoamplifier Enhances CDT (Cellular Level)

The above solution-phase studies demonstrated that the manganese-based CDT nanoamplifier H-MnO_2_/GOX&CQ-iRGD exhibits three critical properties: TME-responsive degradation, controlled drug release, and efficient catalytic activity toward glucose oxidation for H_2_O_2_ generation. Collectively, these features enhance the production of H_2_O_2_ and ∙OH, highlighting the strong potential of this nanoplatform for CDT applications in pancreatic cancer therapy.

**Figure 3:**
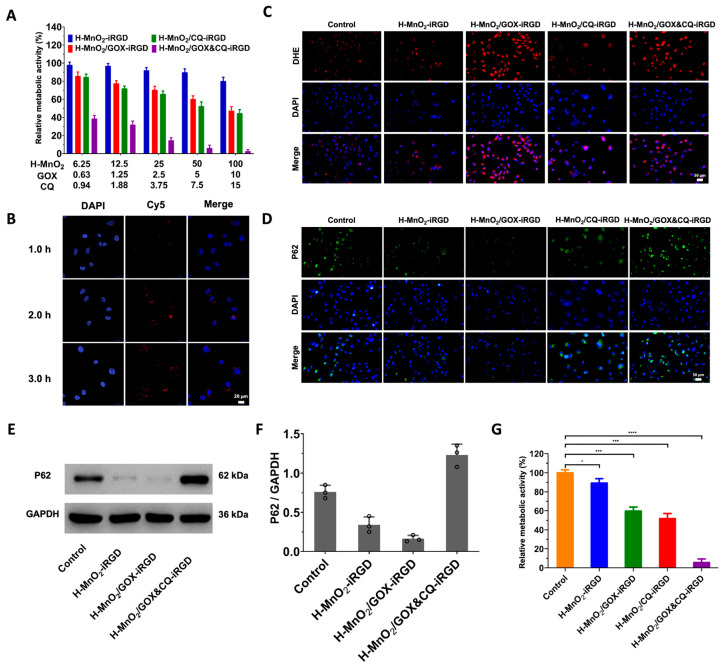
Cellular-level evaluation of H-MnO_2_/GOX&CQ-iRGD in PANC-1 cells. (**A**) Relative metabolic activity of PANC-1 cells after 24 h treatment with H-MnO_2_-iRGD, H-MnO_2_/GOX-iRGD, H-MnO_2_/CQ-iRGD, and H-MnO_2_/GOX&CQ-iRGD. (Concentration unit: μg/mL). (**B**) Confocal fluorescence images of Cy5-labeled H-MnO_2_/GOX&CQ-iRGD uptake at 1, 2, and 3 h (DAPI = blue nuclei; Cy5 = red) (scale bar: 20 μm). (**C**) Intracellular ROS detection by DHE staining under different treatments (λem = 610 nm). (**D**) P62 immunofluorescence for autophagy inhibition following treatment; increased P62 puncta indicate blocked autophagic flux (scale bar: 50 μm). (**E**,**F**) WB analysis of P62 accumulation in PANC-1 cells. (**G**) Comparative metabolism showing synergistic cytotoxicity of the dual-functional nanoplatform. Statistical significance: * *p* < 0.05; *** *p* < 0.001; **** *p* < 0.0001.

To validate these effects at the cellular level, we investigated the in vitro performance of H-MnO_2_/GOX&CQ-iRGD in PANC-1 pancreatic cancer cells, focusing on cytotoxicity, cellular uptake, intracellular H_2_O_2_ generation, and autophagy inhibition. Cells were treated with nanoparticle formulations with or without GOX and CQ for 24 h, followed by apoptosis analysis (Figure 3A). Among all formulations, H-MnO_2_/GOX&CQ-iRGD exhibited the most potent cytotoxicity in a dose-dependent manner. Importantly, even at a concentration of 100 μg/mL, H-MnO_2_-iRGD alone maintained a relative metabolic activity of approximately 80.04%, confirming the intrinsic biocompatibility of the nanocarrier. However, the inclusion of GOX and CQ significantly enhanced the tumor-killing effect, indicating that the dual-loaded system possesses both safety and therapeutic efficacy.

To assess cellular uptake, the nanoparticles were labeled with Cy5 (10 μg/mL) and incubated with PANC-1 cells for 1, 2, and 3 h. Nuclear staining with DAPI was performed to visualize internalization (Figure 3B). After 1 h, faint red fluorescence signals indicated initial uptake of the nanoplatform. With prolonged incubation, intracellular fluorescence intensity increased markedly, confirming efficient internalization over time. This enhanced uptake is attributed to the tumor-targeting and penetrating properties conferred by the iRGD modification.

We next evaluated intracellular H_2_O_2_ generation. ROS levels were measured using the DHE fluorescent probe after treating cells with various nanoparticle formulations (Figure 3C). GOX-containing nanoparticles (H-MnO_2_/GOX-iRGD and H-MnO_2_/GOX&CQ-iRGD) produced significantly elevated ROS levels compared to the control. In contrast, CQ-only particles (H-MnO_2_/CQ-iRGD), lacking GOX, did not significantly alter ROS levels. These results demonstrate that the GOX-mediated oxidation of glucose is crucial for boosting intracellular H_2_O_2_ and enhancing CDT efficacy.

To examine the role of CQ in inhibiting autophagy, we analyzed the accumulation of P62, an autophagy substrate protein that accumulates when autophagic flux is blocked. Immunofluorescence staining and Western blotting of P62 (Figure 3D–F) [42] indicated that CQ-loaded nanoparticles effectively inhibited autophagy, whereas GOX-only formulations did not, highlighting CQ’s key role in blocking oxidative stress-induced autophagy.

Finally, we systematically evaluated the overall cytotoxic efficacy of the nanoplatform using the CCK-8 assay (Figure 3G). Compared with the control and the CDT-only formulation (H-MnO_2_-iRGD), both GOX-loaded (H-MnO_2_/GOX-iRGD) and CQ-loaded (H-MnO_2_/CQ-iRGD) nanoparticles exhibited enhanced tumoricidal effects through distinct mechanisms—H_2_O_2_ augmentation and autophagy inhibition, respectively. Notably, the dual-functionalized H-MnO_2_/GOX&CQ-iRGD formulation achieved the most pronounced cytotoxicity by simultaneously amplifying intracellular oxidative stress and suppressing autophagy. This synergistic mechanism enables the platform to effectively overcome two major bottlenecks of CDT and significantly improve therapeutic outcomes in pancreatic cancer cells.

### 3.4. Cascade Nanoamplifier-Enhanced CDT for Pancreatic Cancer in Animal Models (In Vivo Level)

Encouraged by the promising in vitro results, we further investigated the tumor-targeting capability and therapeutic efficacy of H-MnO_2_/GOX&CQ-iRGD in vivo. Prior to initiating tumor therapy, we first assessed the biosafety of the CDT amplifier. Healthy female nude mice (6 weeks old) were intravenously injected via the tail vein with H-MnO_2_/GOX&CQ-iRGD at a dose of 20 mg/kg. Blood samples and major organs were collected on days 1 and 3 post-injection for analysis. As shown in the hematological profiles (Figure S4), key parameters—including MCH, MCV, HCT, HGB, MCHC, WBC, RDW-CV, RBC, PLT, MPV—remained unchanged compared to pre-injection values. These findings confirm that H-MnO_2_/GOX&CQ-iRGD is well tolerated and exhibits good systemic biosafety.

**Figure 4:**
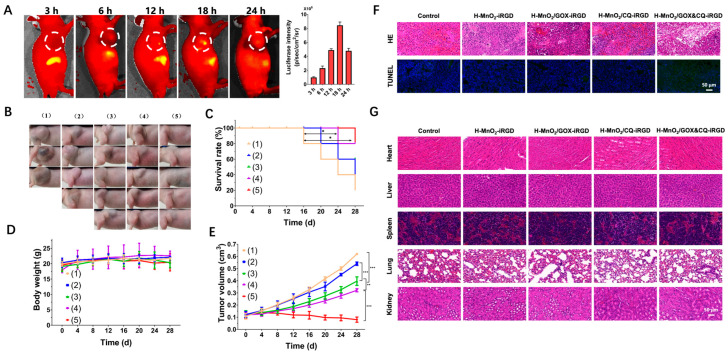
In vivo targeting and therapeutic efficacy of H-MnO_2_/GOX&CQ-iRGD in a pancreatic cancer mouse model. (**A**) Fluorescence imaging and quantitative ROI analysis of Cy7-labeled H-MnO_2_/GOX&CQ-iRGD showing tumor accumulation over 24 h (*n* = 3). (**B**) Representative images of tumor-bearing mice after 20 days of treatment. (**C**) Kaplan–Meier survival curves for each group. (**D**,**E**) Body weight (**D**) and tumor volume (**E**) changes during the 28-day treatment. Tumor volumes were analyzed by two-way ANOVA with Tukey’s post hoc test; survival was analyzed using the log-rank (Gehan–Breslow–Wilcoxon) test. Statistical significance: * *p* < 0.05, ** *p* < 0.01, *** *p* < 0.001. Treatment groups: (1) Control, (2) H-MnO_2_-iRGD, (3) H-MnO_2_/GOX-iRGD, (4) H-MnO_2_/CQ-iRGD, and (5) H-MnO_2_/GOX&CQ-iRGD. (**F**) H&E and TUNEL staining of tumor sections showing extensive necrosis and apoptosis in the dual-loaded group compared to limited effects in control and single-agent groups. (**G**) H&E staining of major organs showing no signs of systemic toxicity.

We further investigated the tumor-targeting capacity and therapeutic efficacy of H-MnO_2_/GOX&CQ-iRGD in vivo using a pancreatic tumor-bearing BALB/c nude mouse model. Mice were intravenously injected with 100 μL of Cy7-labeled H-MnO_2_/GOX&CQ-iRGD (10 mg/mL), and real-time fluorescence imaging was performed at 0, 3, 6, 12, 18, and 24 h post-injection using the LB983 small animal imaging system. As shown in Figure 4A, the nanoplatform initially accumulated in the liver, followed by a gradual and sustained increase in fluorescence signal at the tumor site, peaking at 18 h. These results demonstrate that H-MnO_2_/GOX&CQ-iRGD exhibits excellent tumor-targeting ability via systemic circulation and efficient tumor accumulation, supporting its potential utility in clinical CDT applications.

To evaluate therapeutic efficacy, we randomly assigned PANC-1 subcutaneous model mice of pancreatic tumors to five treatment groups (*n* = 5 per group): (1) saline control, (2) H-MnO_2_-iRGD, (3) H-MnO_2_/GOX-iRGD, (4) H-MnO_2_/CQ-iRGD, and (5) H-MnO_2_/GOX&CQ-iRGD. Tumor volume, body weight, and overall survival were monitored every four days and recorded photographically (Figure 4B). In the control group, tumors grew rapidly, and mortality occurred from day 16 onward due to excessive tumor burden, with only one mouse surviving by the end of this study. The H-MnO_2_-iRGD group showed limited tumor inhibition, likely due to low endogenous H_2_O_2_ levels and unmitigated autophagy. The H-MnO_2_/GOX-iRGD group enhanced intratumoral H_2_O_2_ production via glucose oxidation but was insufficient to suppress tumor growth due to the compensatory activation of autophagy. Conversely, the H-MnO_2_/CQ-iRGD group exhibited effective autophagy inhibition, but the low H_2_O_2_ levels limited CDT efficiency. Only the H-MnO_2_/GOX&CQ-iRGD group displayed a pronounced therapeutic benefit by combining two synergistic mechanisms: (1) GOX-mediated generation of H_2_O_2_ boosted ∙OH production and oxidative mitochondrial damage, and (2) CQ-mediated inhibition of autophagy blocked the tumor’s resistance to oxidative stress. This group exhibited the greatest tumor suppression and significantly improved survival outcomes compared with all other groups. Tumor volumes were analyzed using two-way ANOVA with Tukey’s post hoc test, and survival rates were evaluated using the log-rank (Gehan–Breslow–Wilcoxon) test. Both tumor volume reduction and survival data demonstrated statistically significant differences (Figure 4C–E).

Throughout the treatment period, no significant changes in body weight were observed across the groups. At the conclusion of the 28-day study, surviving animals were sacrificed, and their tumors and major organs (heart, liver, spleen, lung, and kidney) were collected for histological examination, histological analysis of tumor tissues via H&E and TUNEL staining revealed clear differences across groups. In the control and H-MnO_2_-iRGD groups, tumor cells were densely packed and showed no significant signs of necrosis. In contrast, the H-MnO_2_/GOX-iRGD and H-MnO_2_/CQ-iRGD groups displayed evident tumor necrosis, with the most extensive necrotic regions observed in the H-MnO_2_/GOX&CQ-iRGD group (Figure 4F). H&E staining of major organs revealed no signs of systemic toxicity, confirming the biosafety of the nanoplatforms (Figure 4G). In addition, immunohistochemical (IHC) staining was performed on tumor tissues to evaluate the expression of inflammation- and malignancy-associated markers, including CD68, MCP-1, MPO, and TGF-β. The control and H-MnO_2_-iRGD groups showed the highest expression levels of all markers, indicating severe tumor-associated inflammation and poor therapeutic response. Moderate reduction in marker expression was observed in the H-MnO_2_/GOX-iRGD and H-MnO_2_/CQ-iRGD groups, reflecting partial therapeutic efficacy. Notably, the H-MnO_2_/GOX&CQ-iRGD group exhibited the lowest expression of all markers, consistent with the strongest antitumor effect (Figure S5). These findings affirm that the H-MnO_2_/GOX&CQ-iRGD nanoplatform not only achieves high tumor specificity and biocompatibility, but also exerts potent antitumor effects in vivo, effectively inhibiting pancreatic cancer progression.

## 4. Conclusions

Pancreatic cancer remains one of the most challenging malignancies due to the lack of effective therapeutic options, underscoring the urgent need for innovative strategies. We developed a TME-responsive CDT nanoamplifier—H-MnO_2_/GOX&CQ-iRGD—based on hollow MnO_2_ nanostructures. This system co-delivers GOX to elevate H_2_O_2_ production in situ and CQ to inhibit autophagy, while iRGD peptide modification enhances tumor penetration through the dense stromal matrix. Under acidic conditions, MnO_2_ decomposes to release Mn^2+^ and O_2_, relieving hypoxia and catalyzing ∙OH generation. Concurrently, CQ suppresses autophagy, amplifying oxidative damage. This integrated ‘targeting–dissociation–catalysis–inhibition’ strategy demonstrated potent antitumor activity, excellent biosafety, and high tumor selectivity in both in vitro and in vivo models. offering a translationally promising platform for CDT-based pancreatic cancer therapy.

## Data Availability

The original contributions presented in this study are included in this article/Supplementary Material. Further inquiries can be directed to the corresponding authors.

## Supplementary Materials

The following supporting information can be downloaded at: https://www.mdpi.com/article/10.3390/pharmaceutics17091201/s1, Figure S1: Synthesis and characterization of hollow MnO_2_ nanostructures; Figure S2: Feasibility evaluation of drug GOX&CQ loading on H-MnO_2_; Figure S3:Drug release of H-MnO_2_/GOX&CQ-iRGD in different simulated environments; Figure S4: Biocompatibility testing of H-MnO_2_/GOX&CQ-iRGD; Figure S5: Histological evaluation of subcutaneous tumor model treatment: Immunohistochemical staining for CD68, MCP-1, MPO, and TGF-β in tumor tissues.

## Abbreviations

The following abbreviations are used in this manuscript:

GOX	glucose oxidase
CQ	chloroquine
TME	tumor microenvironment
CDT	chemodynamic therapy
GSH	glutathione
PEI	polyethyleneimine
ROS	reactive oxygen species
BPEI	branched polyethyleneimine
