# Supplementary material for: Pancreatic Cancer-Targeting Cascade Nanoamplifier Enables Self-Replenishing H2O2 Generation and Autophagy Disruption in Chemodynamic Therapy
Source: Pharmaceutics. 2025 Sep 16;17(9):1201. doi: 10.3390/pharmaceutics17091201 (PMC12473349; doi:10.3390/pharmaceutics17091201)
Supplement: Supplementary file 1 [file pharmaceutics-17-01201-s001.zip › pharmaceutics-3784897-supplementary.pdf]

Supporting Information

Pancreatic Cancer-Targeting Cascade Nanoamplifier Enables Self-Replenishing H_2O_2 Generation and Autophagy Disruption in Chemodynamic Therapy

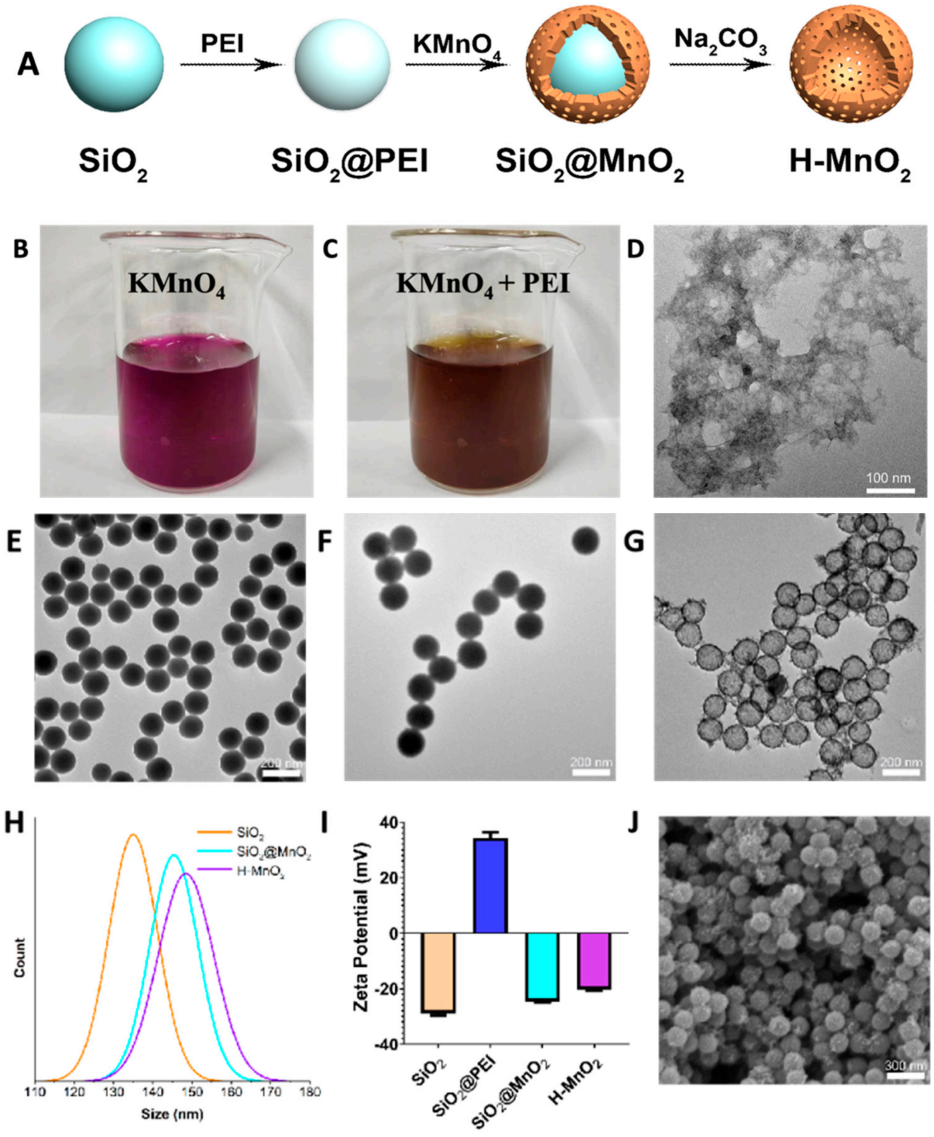

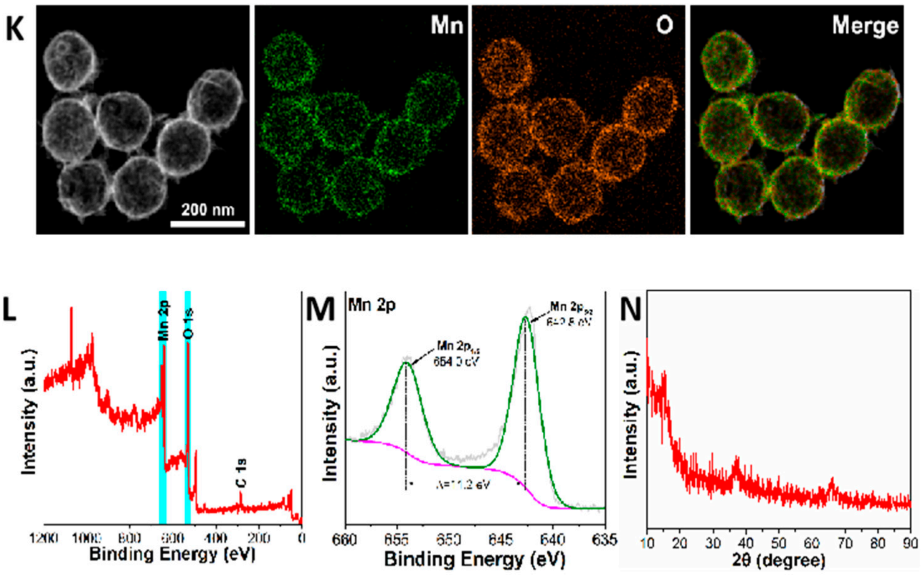

Figure S1. Synthesis and Characterization of Hollow MnO_2 Nanostructures. (A) Schematic of the H-MnO_2 synthesis process. (B–C) Color change of $KMnO_4$ solution before and after PEI reduction.(D) TEM image of MnO_2 synthesized via PEI-mediated $KMnO_4$ reduction.(E-G) The TEM diagram of SiO_2 (E), SiO_2@MnO_2 (F), and H-MnO_2 (G). (H) The corresponding particle size distribution diagram. (I) The zeta potential of SiO_2, SiO_2@PEI, SiO_2@MnO_2, and H-MnO_2. (J) SEM diagram of H-MnO_2.(K) Elemental mapping confirming uniform Mn and O distribution. (L) XPS survey spectrum of H-MnO_2. (M) High-resolution Mn 2p XPS spectrum indicating Mn(IV). (N) XRD pattern demonstrating amorphous structure.

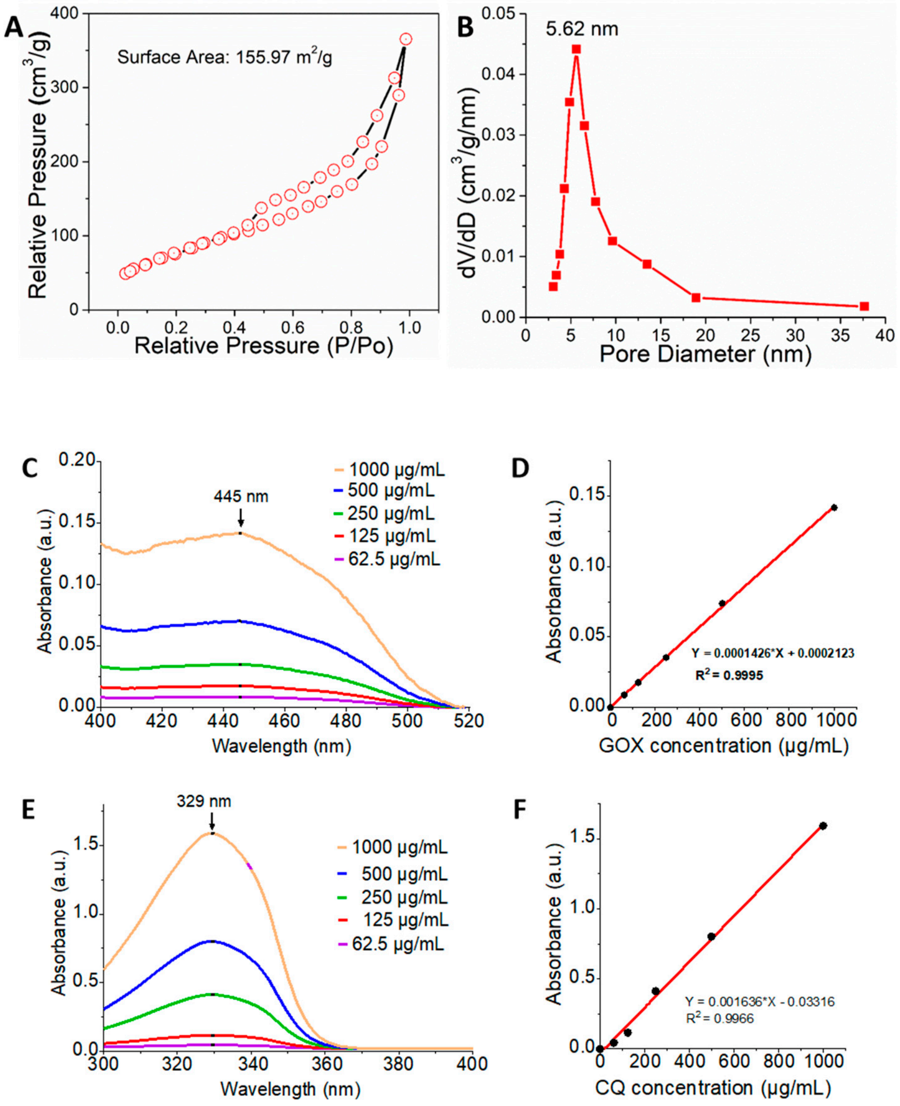

Figure S2. Feasibility evaluation of drug GOX&CQ loading on H-MnO_2。 Stepwise Construction and Surface Functionalization of H-MnO_2/GOX&CQ-iRGD Nanoamplifier.(A) N_2 adsorption desorption isotherm and (B) pore size distribution of H-MnO_2.(C) UV–vis absorption spectra of GOX solutions at increasing concentrations (0–1000 μg/mL). (D) Corresponding calibration curve of GOX absorbance at 445 nm versus concentration. (E) UV–vis absorption spectra of CQ solutions at various concentrations (0–1000 μg/mL). (F) Corresponding calibration curve of CQ absorbance at 329 nm versus concentration.

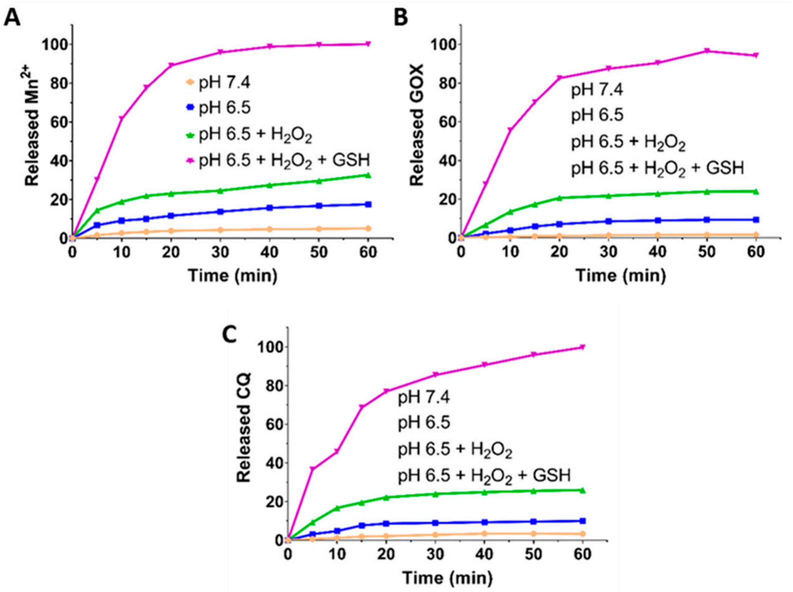

Figure S3. Drug release of H-MnO_2/GOX&CQ-iRGD in different simulated environments. Release rates of Mn^{2+} (A), GOX (B), and CQ (C) from H-MnO_2/GOX&CQ-iRGD in solutions with pH 7.4, pH 6.5, pH 6.5 + H_2O_2, and pH 6.5 + H_2O_2+ GSH (GSH: 5 mM, H_2O_2: 100 μM).

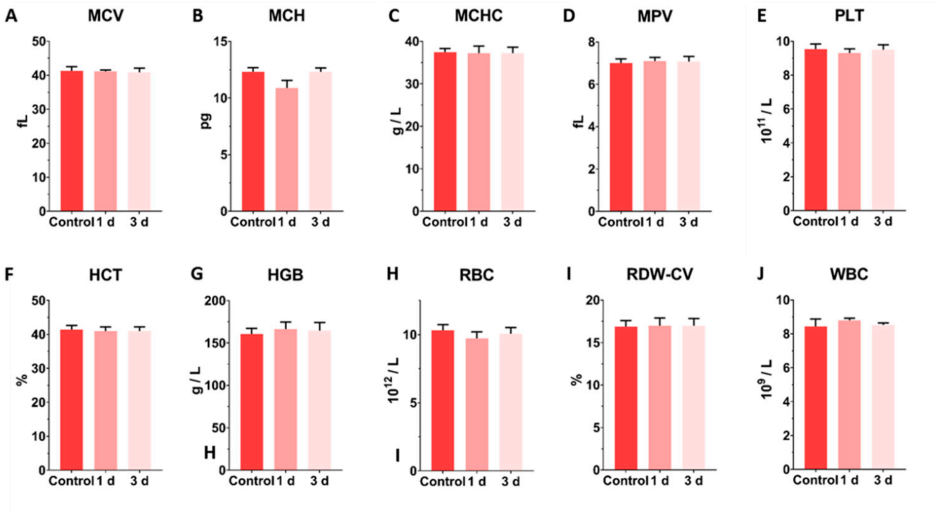

Figure S4. Biocompatibility testing of H-MnO_2/GOX&CQ-iRGD.(A) Mean corpuscular volume (MCV), (B) Mean corpuscular hemoglobin (MCH), (C) Mean corpuscular hemoglobin concentration (MCHC), (D) Mean platelet volume (MPV), (E) Platelet (PLT), (F) Hematocrit (HCT), (G) Hemoglobin (HGB), (H) Red blood cell (RBC), (I) Red blood cell distribution width variation coefficient (RDW-CV), and (J) White blood cell (WBC).

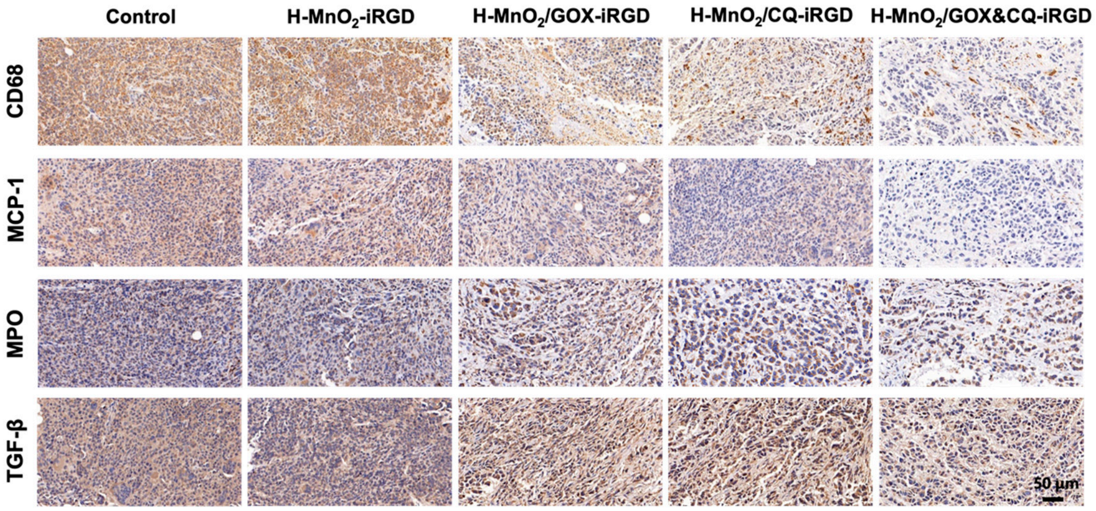

Figure S5. Histological evaluation of subcutaneous tumor model treatment: Immunohistochemical staining for CD68, MCP-1, MPO, and TGF-β in tumor tissues.
